# An enhanced speech emotion recognition using vision transformer

**DOI:** 10.1038/s41598-024-63776-4

**Published:** 2024-06-07

**Authors:** Samson Akinpelu, Serestina Viriri, Adekanmi Adegun

**Affiliations:** School of Mathematics, Statistics and Computer Science, University of KwaZulu-Natal, Durban, 4001 South Africa

**Keywords:** Human–computer interaction, Deep learning, Speech emotion recognition, CNN, Vision transformer, Mel spectrogram, Mathematics and computing, Computer science

## Abstract

In human–computer interaction systems, speech emotion recognition (SER) plays a crucial role because it enables computers to understand and react to users’ emotions. In the past, SER has significantly emphasised acoustic properties extracted from speech signals. The use of visual signals for enhancing SER performance, however, has been made possible by recent developments in deep learning and computer vision. This work utilizes a lightweight Vision Transformer (ViT) model to propose a novel method for improving speech emotion recognition. We leverage the ViT model’s capabilities to capture spatial dependencies and high-level features in images which are adequate indicators of emotional states from mel spectrogram input fed into the model. To determine the efficiency of our proposed approach, we conduct a comprehensive experiment on two benchmark speech emotion datasets, the Toronto English Speech Set (TESS) and the Berlin Emotional Database (EMODB). The results of our extensive experiment demonstrate a considerable improvement in speech emotion recognition accuracy attesting to its generalizability as it achieved 98%, 91%, and 93% (TESS-EMODB) accuracy respectively on the datasets. The outcomes of the comparative experiment show that the non-overlapping patch-based feature extraction method substantially improves the discipline of speech emotion recognition. Our research indicates the potential for integrating vision transformer models into SER systems, opening up fresh opportunities for real-world applications requiring accurate emotion recognition from speech compared with other state-of-the-art techniques.

## Introduction

Human–computer interactions (HCI) can be improved by paying more attention to emotional cues in human speech^1^. The need for speech recognition and enhancement of emotion recognition in achieving more natural interaction and better immersion is becoming more of a challenge as a result of the growing trend in artificial intelligence (AI)^2,3^. Coincidentally, with the development of deep neural networks, research on Speech Emotion Recognition (SER) systems has grown steadily by turning audio signals into feature maps that vividly describe the vocal traits of speech(auditory) samples.^4^.

**Figure 1:**
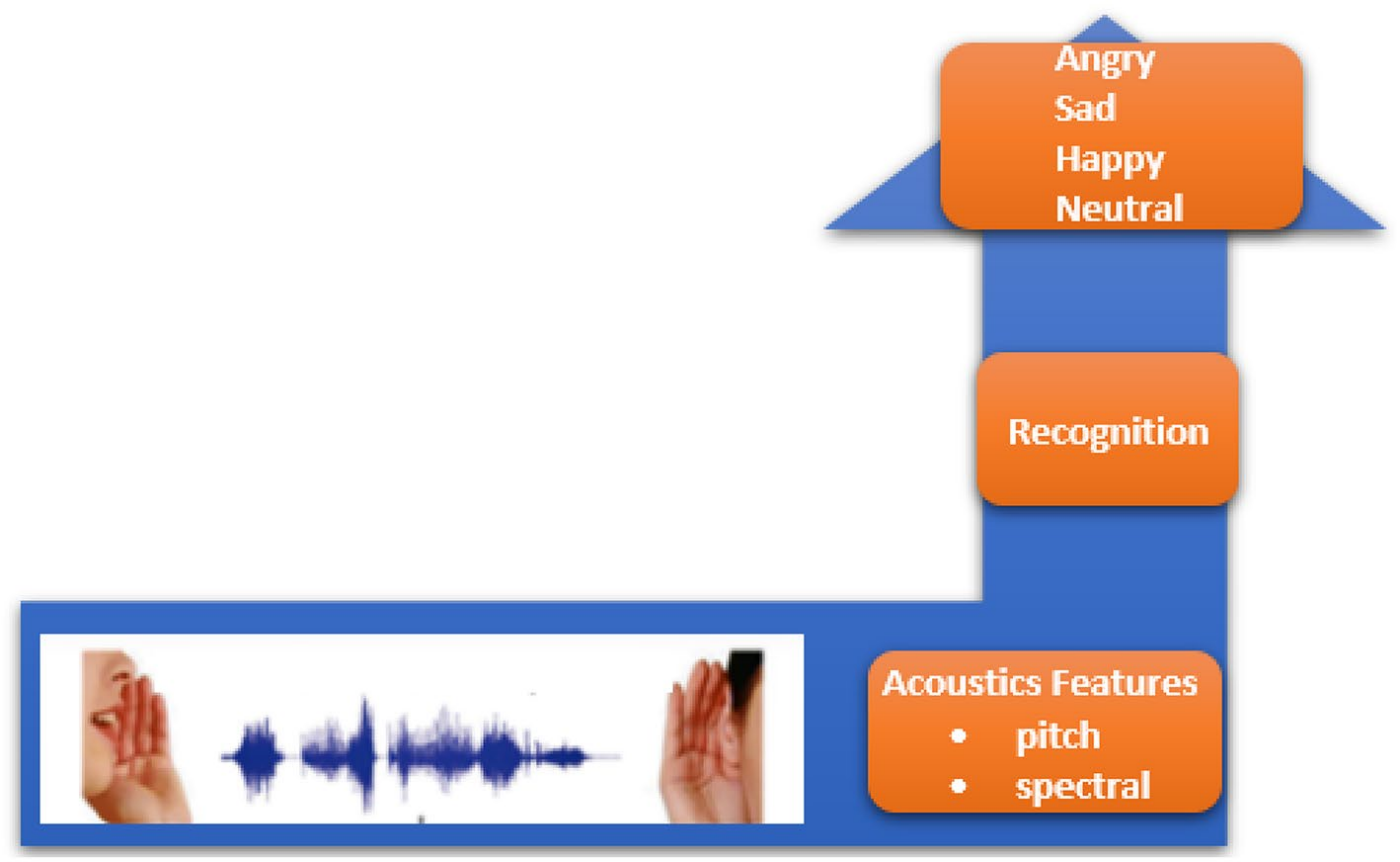
Traditional speech emotion recognition framework.

Speech Emotion Recognition (SER) is a classification problem that seeks to classify audio samples into pre-defined emotions. SER has applications in affective computing, psychological wellness evaluation, and virtual assistants, and has become a crucial field of research in human–computer interaction^5^. Speech signals may be used to reliably detect and comprehend human emotions, which enables machines to react correctly and produce more interesting and tailored interactions^6^. By acquiring acoustic features from speech signals^7^, such as pitch, energy, and spectral qualities, and using machine learning algorithms to categorize emotions based on these features, has been the concentration of conventional approaches (Fig. 1) to SER^8^. Although these methods have yielded encouraging results, they frequently fail to pick up on nuances in emotional cues and are subject to noise and unpredictability in voice signals.

Researchers have been able to improve SER by using the spectral features of an audio sample as an image input to the impressive advancements in computer vision. Convolutional neural networks (CNNs), in particular, have shown astounding performance in deep learning^9^ models for visual tasks like image processing and object detection. The weights of several convolutional layers have been utilized to create feature representations in this architecture^10,11^. Utilizing mel-spectrograms, this method can be used in SERs to convert audio data into visual audio signals based on its frequency components. Then, these representations that resemble images can be trained using a CNN network. Traditional CNN, however, only accepts a single frame as input and does not compute over a timestep sequence, therefore they are unable to remember previous data from the same sample while processing the subsequent timestamp.

Additionally, because of the number of parameters generated by the numerous convolutional layers, they provide large levels of computational complexity^12^. Researchers have been seeking alternative architectures that are more appropriate for handling visual data in the context of SER as a result of this constraint.

The Vision Transformer (ViT) is one such architectural design that has attracted significant interest. The ViT model, which was initially introduced for image classification tasks, completely changed the area of computer vision by exhibiting competitive performance without utilizing conventional CNN building blocks^13^. The ViT model makes use of a self-attention mechanism that enables it to directly learn global features from the input image and capture spatial dependencies. This unique model has demonstrated promising performance in several computer vision applications^14^, raising the question of whether it may be leveraged to enhance SER.

In this study, we addressed two core issues. At first, the computational complexity is reduced, we enhanced the accuracy of emotion recognition from speech signals by improving the state-of-the-art performance. We focus mainly on extracting features from the mel-spectrogram^15^ and fed it into a novel lightweight ViT model with a self-attention mechanism for accurate speech emotion recognition. The spectrogram image is represented in time and frequency as width and length to enable our proposed model to learn emotionally rich features from speech signals. Computational cost is reduced as a result of fewer over-blotted parameters. The major contributions of this work are highlighted below.

- We proposed a novel lightweight Vision Transformer (ViT) model with self-attention for learning deep features related to emotional cues from the mel-spectrogram to recognize emotion from speech.
- Complexity is reduced for SER through fewer parameters and efficient layers that learn discriminative features from the input image.
- We evaluated the proposed SER model on popular benchmark datasets which include TESS and EMO-DB. The result of our comparative experiments shows an improved performance in SER, confirming its suitability for real-time application.

The remaining part of this paper is split into other sections as follows. Section 2 presents the reviewed literature and related works, Section 3 highlights the proposed methodology and its detailed description. In Section 4, the experimental configuration, result and discussion are presented, while Section 5 illustrates the conclusion and future work to foster research progress in the SER domain.

## Review of related works

The study of emotion recognition from speech signals as it plays a crucial role in behavioural patterns and enhances human–computer interaction in the past decade has come a long way. Identification of human emotional conditions from speech samples (natural or synthetic) has formed the basis for the development of Speech Emotion Recognition SER systems. Core among these emotional states are angry, sad, happy, neutral, etc. Researchers began with the conventional approach of recognizing these emotions with the use of orthodox machine learning models which includes Support Vector Machine(SVM)^16–18^, Gaussian Mixture Model(GMM)^19^,k-nearest Neighbour(KNN)^20^ and Hidden Markov Model (HMM)^21^ among others. However, these classical machine learning classifiers are bewildered with the problem of high susceptibility to noise and the inability to efficiently handle large audio speech samples.

Therefore, neural network approaches such as Recurrent Neural Networks (RNN)^22^ and Long Short Term Memory (LSTM)^23–25^ have been proposed by researchers in the SER domain, because of their capability to handle sequence(time series) data and learn temporal information that is critical to emotion recognition using contextual dependencies. The adoption of these two techniques has littered several SER literature, because emotion recognition has been improved upon. However, RNN is prone to gradient descent problems^26^

The common approach to SER in recent came as a result of unimaginable success through deep learning techniques^27,28^ and prominent among this approach are Convolutional Neural Networks (CNN)^29^, Deep Neural Networks(DNN)^30–32^, Deep Belief Networks(DBN)^33^ and Deep Boltzman Machine (DBM)^34^. In Zeng et al.^35^ spectrogram feature extracted from Rayson Audio-Visual Database of Emotional Speech and Song(RAVDESSS) speech dataset was fed into DNN with gated residual network which yielded 65.97% accuracy of emotion recognition on tested data. In the same vein, a pre-trained VGG-16 convolutional neural network was utilized in Popova et al.^36^ and they achieved an accuracy of 71% after extensive experiments. To increase the possibility of improving the recognition rate, the author Issa et al.^37^ proposed a novel Deep Convolutional Neural Network (DNN) for SER. Multiple features Similarlyrances were extracted such as Mel Frequency Cepstral Coefficient, spectral contrast, and Mel-Spectrogram, and were fused to serve as their model input. Their method arrived at 71.61% accuracy for recognising eight different emotions from the RAVDESS dataset. Their method was also experimented on EMODB and IEMOCAP datasets for generalizability. However, their CNN model could not efficiently capture the spatial features and sequences peculiar to speech signals. In addressing the foregone, a multimodal approach of deep learning and temporal alignment techniques was proposed by Li et al.^38^. In their method, CNN, LSTM and Attention Mechanism were combined and they achieved the highest accuracy of 70.8% with semantic embeddings.

In recent times as well, the combination of CNN, LSTM or RNN for SER tasks has recorded significant improvement^39^. This approach relies heavily on the extraction of features from raw speech signals with CNN and passing them into the LSTM or RNN for extraction of long-term dependencies features that are peculiar to emotion recognition from auditory utterances^40^. Puri et al.^41^ implemented a hybrid approach of utilizing LSTM and DNN on the RAVDESS dataset. They extracted MFCC from raw speech signals and fed it into their model. The ensemble technique of extracting salient features from speech utterances and passing the emotional features into a classifier, irrespective of the language and cultural background of the speakers has also aroused the interest of researchers in the SER field. High-level features from speech signals were extracted using DBN and then later fed into a Support Vector Machine classifier for emotion classification in Schuller et al.^42^. Similarly, Zhu et al.^43^ utilized DNN and SVM and experimented with the efficiency of their model on the Chinese Academy of Chinese-based dataset. A separate study by Pawar et al.^44^ proposed a deep learning approach for SER. Relevant features were extracted from speech signals using MFCC, as input to train the CNN model. They achieve a significant result of 93.8% accuracy on the EMODB dataset. The author in^45^ proposed innovative lightweight multi-acoustic features-based DCNN techniques for speech emotion recognition. In their method, various features such as Zero Crossing Rate(ZCR), wavelet packet transform (WPT), spectral roll-off, linear prediction cepstral coefficients (LPCC), pitch, etc. were extracted and fed into one-dimensional DCNN and they obtained 93.31% on Berlin Database of Emotional Speech(EMODB) and 94.18% on RAVDESS respectively. Badshah et al.^46^ presented present a double CNN-based model for SER with spectrogram from an audio signal. They utilized a pooling mechanism and kernel of different sizes with spectrogram input generated using Fast Fourier Transform (FFT). Their approach validates the importance of max-pooling operation in CNN.

The introduction of audio transformer to speech paralinguistics has contributed immensely to emotion recognition from speech signals. It involves analysis and synthesis of speech signals with features that are non-verbal^47^. Chen et al^48^ proposed a novel full-stack audio transformer (WavLM) for speech analysis using a speech denoising approach for learning general speech representations from huge unannotated data. The performance of their proposed transformer model, benchmarked on the SUPERB dataset achieved a state-of-the-art result and improved many speech-related tasks such as speech emotion recognition and speaker verification or identification. Xu et al.^49^ proposed a novel speech transformer-based that incorporated self-attention and local dense synthesizer attention (LDSA) for extracting both local and global features from speech signals. In a bid to enhance the efficiency of end-to-end speech recognition models while lowering computing complexity, the technique eliminates pairwise interactions and dot products and limits attention scope to a narrow region surrounding the current frame. A novel hybrid-based audio transformer, named Conformer-HuBERT was implemented by Shor et al.^50^. Their mechanism achieves a significant milestone in emotion recognition from speech signals and other paralinguistic tasks by learning from many large-scale unannotated data. Again, Chen et al.^51^ proposed a novel SpeechFormer technique that combines the distinctive features of speech signals into transformer models. A hierarchical encoder that uses convolutional and pooling layers to shorten the input sequence is one of the three components of the framework. Another is a local self-attention module that records dependencies inside a predetermined window size, and a global self-attention module that records dependencies across various windows. Paraformer is another novel speech transformer model for non-autoregressive end-to-end speech recognition that employs parallel attention and parallel decoder approaches, introduced by Gao et al.^52^. The framework enables independent prediction of each output token without reliance on prior output tokens, and permits each decoder layer to handle all encoder outputs concurrently without waiting for previous decoder outputs. The study demonstrates that Paraformer achieves faster inference speed and higher accuracy on multiple speech recognition datasets compared to existing non-autoregressive models.

In the immediate past, efforts towards improving the efficiency of deep learning model performance and conquering the challenge of long-range dependencies peculiar to the CNN-base model for SER have been increased. The state-of-the-art transformer model has been introduced into SER^53^. A parallel architecture that utilized the ResNet and Transformer model was proposed in Han et al.^54^. Vijay et al.^55^ implemented an audio-video multimodal transformer for emotion recognition. They adopted three self-attention and block embedding to capture relevant features from spectrogram images. Their model achieved 93.59%, 72.45%, and 99.17% on RAVDESS, CREMA-D and SAVEE datasets respectively, but huge computing resources were required because of the architecture. Not quite long after, Slimi et al.^56^ proposed a transformer-based CNN for SER, with hybrid time distribution. They leverage the superior capability of the transformer and achieve a promising result of 82.72% accuracy. However, such a model is prone to high computational complexity due to huge parameters. The ability of CNN-based models to recognize long-range dependencies in speech signals is constrained by the fact that they frequently operate on fixed-size input windows. Speech emotion frequently displays temporal dynamics outside of the speech sequence’s local regions. Therefore, we proposed a lightweight Vision Transformer (ViT) model comprised of a self-attention mechanism^57^ that enables it to capture global contextual information, making it possible to model long-range dependencies and enhance the representation of emotional speech patterns, hence improving speech emotion recognition.

Additionally, while a couple of research studies have looked at how to include visual cues in speech emotion recognition, they frequently treat visual and auditory modalities independently, resulting in an insufficient fusion of information or features. This study seeks to leverage the synergistic effects of multimodal information, enabling a more thorough comprehension of emotions and enhancing the accuracy of the SER system by using the ViT model^58,59^, capable of capturing salient features from the speech signal.

## Proposed method

**Figure 2:**
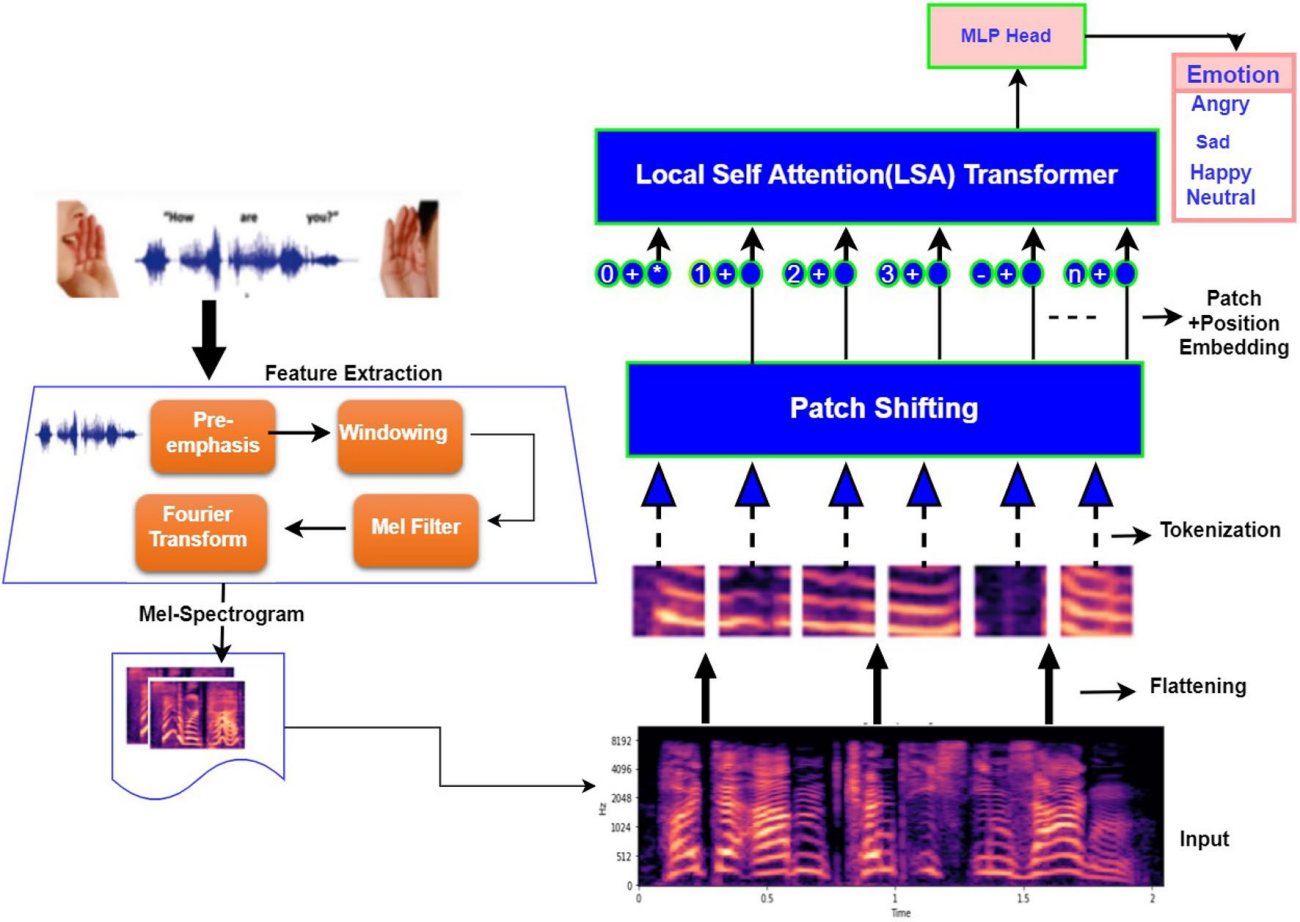
Propose Vision Transformer Architectural Framework.

In this section, we delve into the overview of our proposed model (Fig. 2) for SER. We highlighted the overall details from speech collection, pre-processing, feature extraction, and feeding of ViT with feature vectors that eventually lead to emotion recognition.

### Speech pre-processing

When background noise cannot be tolerated, pre-processing the speech sound is a crucial step. These systems, such as speech emotion recognition (SER) require effective feature extraction from audio files, where the majority of the spoken component consists of salient characteristics connected to emotions. This study used pre-emphasis and silent removal strategies to reach its goal^60^. Pre-emphasis uses Eq. (1) to increase the high-frequency parts of speech signals. The pre-emphasis technique can improve the signal-to-noise ratio by enhancing high frequencies in speech while leaving low frequencies untouched through the Finite impulse response (FIR) mechanism.

1 $$\begin{aligned} H(z) = 1- \alpha {z^{-1}}, \alpha = [1, -0.97] \end{aligned}$$

where *z* is the signal and $\alpha$ is the energy level change across the frequency

Contrariwise, Eq. (2) is used in signal normalization to ensure that speech signals are equivalent despite any differences in magnitude.

2 $$\begin{aligned} S_{Ni} = \frac{S_i-\mu }{\sigma } \end{aligned}$$

where the signal’s mean and standard deviation are represented by $\mu$ and, $\sigma$ respectively, while the signal’s $i^th$ portion is denoted by the $S_i$. The normalized $i^th$ component of the signal is referred to as $SN_i$.

### Extraction of mel-spectrogram Feature

The quality of the feature set heavily influences the recognition performance of the model. As a result, inappropriate features could produce subpar recognition outcomes. To achieve acceptable recognition performance in the context of Deep Learning (DL), extracting a meaningful feature set is a vital task. According to^61^, feature extraction is a crucial step in deep learning since the SER model’s success or failure depends heavily on the variability of the features it uses to do the recognition task. If the derived traits have a strong correlation with the emotion class, recognition will be accurate, but if not, it will be challenging and inaccurate. The performance of recognition in SER is strongly influenced by the quality of the feature set.

**Figure 3:**
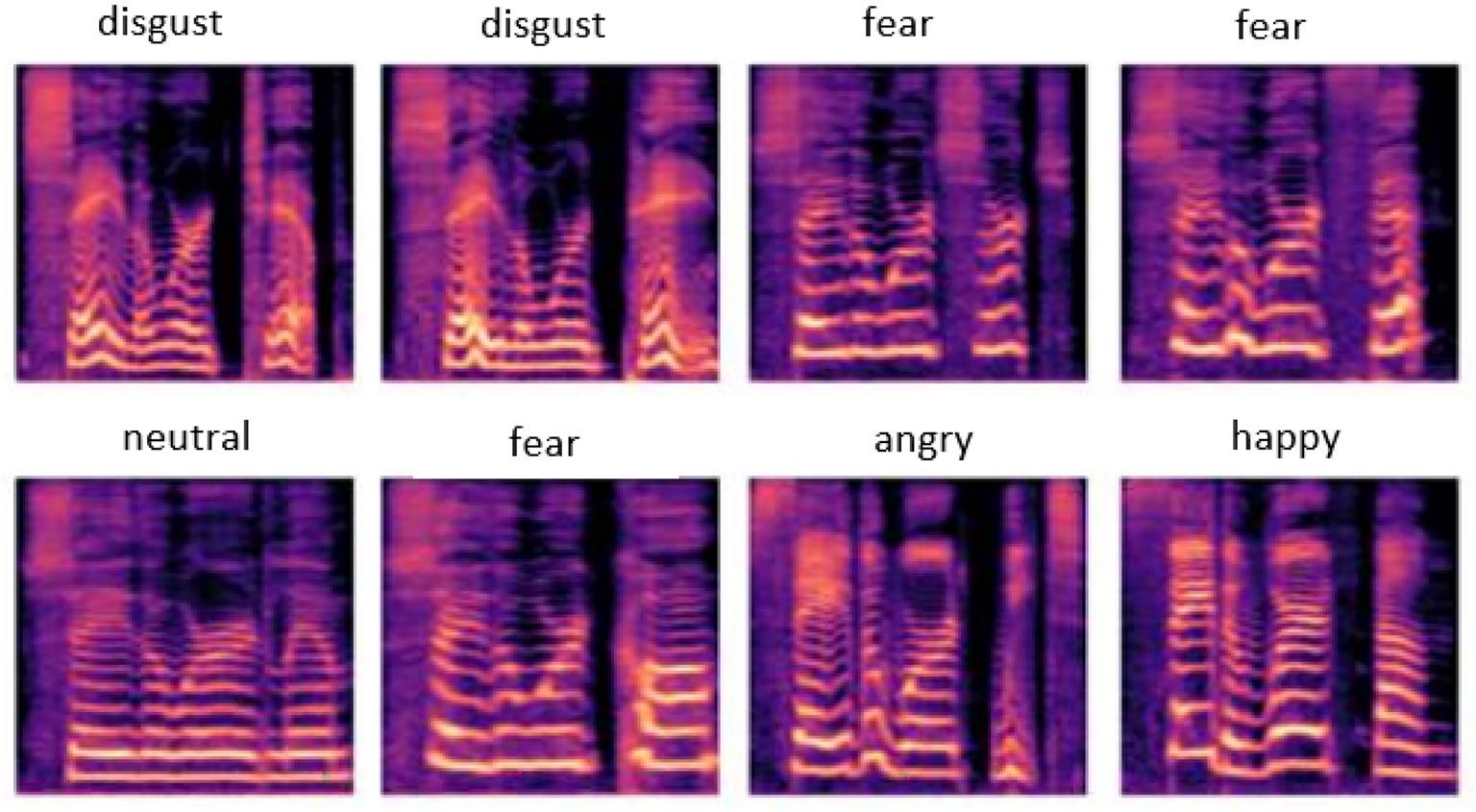
Mel-spectrogram of selected emotion.

The process of mel-spectrograms (Fig. 3) feature extraction involves pre-emphasis, framing, windowing and the discrete Short Time Fourier Transform. In our method, we generate a mel-spectrogram image by converting each speech sound sample into a 2D time-frequency matrix. We perform the discrete Short-Time Fourier Transform (STFT) computation for this. We employ an STFT length of 1024, hop size of 128, and 1024 window size (using Hanning as the window function). Additionally, we used 128*Mel* bins to map the frequency onto the Mel scale. Each audio sound was split into frames of 25 ms, with a 10 ms gap between each frame, to avert information degradation. After the framing and windowing, we applied several mel-filter banks and the mel denotes the ears’ perceived frequency, which is computed using Eq. 3.

3 $$\begin{aligned} Mel(f) = 295 \times \log _{10}\left( {1+\frac{f}{700}}\right) \end{aligned}$$

where *f* represent the real frequency and *Mel*(*f*) represent the corresponding frequency of perception.

### Vision transformer

Vision transformers are becoming the standard in the NLP (Natural Language Processing) domain. The attention mechanism is an important element of such a model. It may extract useful features from the input using a typical query, key, and value structure, where the similarity between queries and keys is pulled out by matrix multiplication between queries and keys. In order to effectively extract many scales, multiple resolutions, and high-level spatial features, vision transformers use a multi-head attention mechanism. The global average pooling system is then used to up-sample and concatenate the dense feature maps that have been produced. To be able to successfully learn and extract the intricate features relevant to emotion recognition in mel-spectrogram image, the method makes use of both local and global attention, as well as global average pooling. As illustrated in our proposed architecture, the entire model ranges from flattening to the classification of emotion. The input image is broken up into patches of defined size, fattened and linearly embedded, added to position embedding, and then transferred to the Transformer encoder.

The Vision Transformers have much less image-specific inductive bias than CNNs, hence, we leverage its capability to classify seven human emotions: angry, sad, disgust, fear, happiness, neutral and surprise as shown in our model. Our proposed vision transformer model for SER is not heavy, unlike many baseline models. It comprises 4, 166, 151 total and trainable parameters, with 0 non-trainable parameters, thereby it reduces computational complexity. In the first stage, a feature vector of shape $(n+1, d)$ is created by embedding an input image(spectrogram) of shape (height, width, and channels) into it^62^. Then, in raster order, the image is splatted into *n* square patches of shape (*t*, *t*, *c*), where *t* is a pre-defined value. Patches are then flattened, producing n line vectors with the shape $(1, t^2*c)$. The flattened patches are multiplied by a trainable embedding tensor of shape $(t^2*c, d)$ that learns to linearly project each flat patch to dimension d. Our model dimension is 128, with 32 patch sizes.

The ViT model’s functional components and corresponding functions in the model architecture are succinctly summarized by the functional components as shown in Table 1. Collectively, they improve the ViT model’s ability to identify spatial dependencies and extract relevant representations from speech signals for recognition of speech emotions.

**Table 1:** Functional Components of the ViTSER.

SN	Components	Description
1	Patch Embeddings	The initial representation of linearly projected image patches with each patch having a vector representation.
2	Positional Enconding	Provides the input embeddings positional information, which enables the model to comprehend the spatial relationship between several patches.
3	Transformer Encoder	Comprised of feedforward neural network modules and several layers of self-attention that capture high-level features and long-range dependencies from the speech signal.
4	Self-Attention	A method for capturing the dependencies among the various patches in the input sequence which enables the model to focus on relevant information across the whole input sequence.
5	Layer-Normalization	Stabilizes training and enhances generalization by normalizing each layer’s activations.
6	Dropout	Regularization method that, during training, randomly sets a portion of the input units to zero thereby, increases the robustness of the model and helps avoid overfitting.

#### Core module analysis of ViT

The proposed ViTSER model in this study utilizes two core audio transformer modules which are self-attention and multi-head attention. The first mechanism is self-attention, which computes representations for the inputs by relating various positions of input sequences. It employs three specific inputs: values (*V*), keys (*K*), and queries (*Q*). The result of a single query is calculated as the weighted sum of the values, with each weight being determined by a specially constructed query function that uses the associated key. Here, we employ an efficient self-attention method that is based on Dot-product^63^as computed in Eq. 4.

4 $$\begin{aligned} Attention({\textbf {Q, K, V}})= softmax(\frac{QK^T}{\sqrt{d_k}}){\textbf {V}} \end{aligned}$$

where the softmax function is prevented from entering regions with extremely small gradients by using the scalar $\frac{1}{\sqrt{d_k}}$.

Secondly, another core module of the audio transformer is multi-head attention, which is used to simultaneously exploit several attending representations. The calculation of multi-head attention is *h* times scaled Dot-Product Attention, where *h* is the number of heads. Three linear projections are used before each attention for transforming the queries, keys, and values, respectively, into more discriminating representations. Next, as shown in Eq. 5, each Scaled Dot-Product Attention is computed separately and its outputs are concatenated.

5 $$\begin{aligned} MultiHead(Q,K,V) = Concat(head_1,..., head_h){\textbf {W}}^O \end{aligned}$$

where $$ head_i=Attention({\textbf {QW}}^{Q}_{i}, {\textbf {KW}}^{K}_{i}, {\textbf {VW}}^{V}_{i})$$

We employed an activation function known as Gaussian Error Linear Unit (GELU), a high-performing activation function in many speech-related tasks and NLP^64^ as compared to RELu (Reactivation Linear Unit). Rather than gating inputs by their sign as in ReLUs, the GELU non-linearity weights inputs according to their value. The GELU activation function is $x\Phi (x)$, for an input *x* is defined from Eq. 6.

6 $$\begin{aligned} GELU(x) =x\Phi (x)= x.\frac{1}{2}\left[ 1+erf(x/\sqrt{2} \right] \end{aligned}$$

where $\Phi (x)$ denotes the standard Gaussian cumulative distribution function.

## Experimental result

In this section, the full details of how we carried out our extensive experiment and evaluation of our model are highlighted. To demonstrate the significance and robustness of our model for the SER utilizing speech spectrograms, we effectively validate our system in this part using two benchmark TESS and EMODB speech datasets. Using the same phenomena, we evaluated the effectiveness of our SER system and contrasted it with other baseline SER systems. The next sections go into further detail on the datasets that were used, the accuracy matrices, and the results of the study.

### Datasets

#### TESS

**Figure 4:**
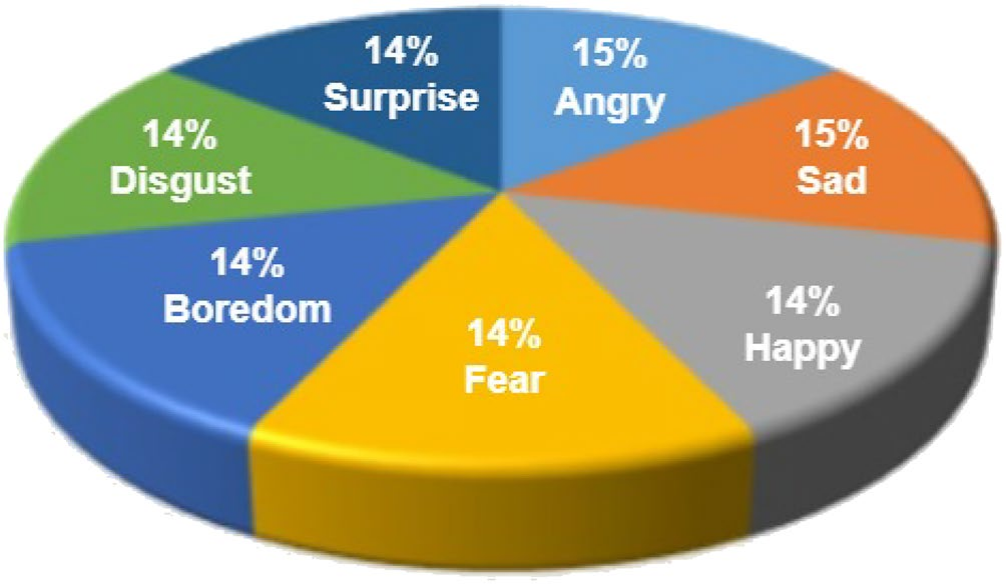
TESS dataset emotion distribution.

The Toronto English Speech Set, or TESS for short, one of the largest freely available datasets, has been used in numerous SER projects. The Auditory Laboratory at Northwestern University recorded TESS speech samples in 2010^65^. During the spontaneous event, two actors were given instructions to pronounce a couple of the 200 words. Their voices were recorded, providing a comprehensive collection of 2800 speech utterances. Seven different feelings were seen in the scenario: happy, angry, scared, disgusted, pleasant, surprised, sad, and neutral. Figure 4 provides an illustration of the TESS description based on each emotion’s contribution to the whole speech dataset.

**Figure 5:**
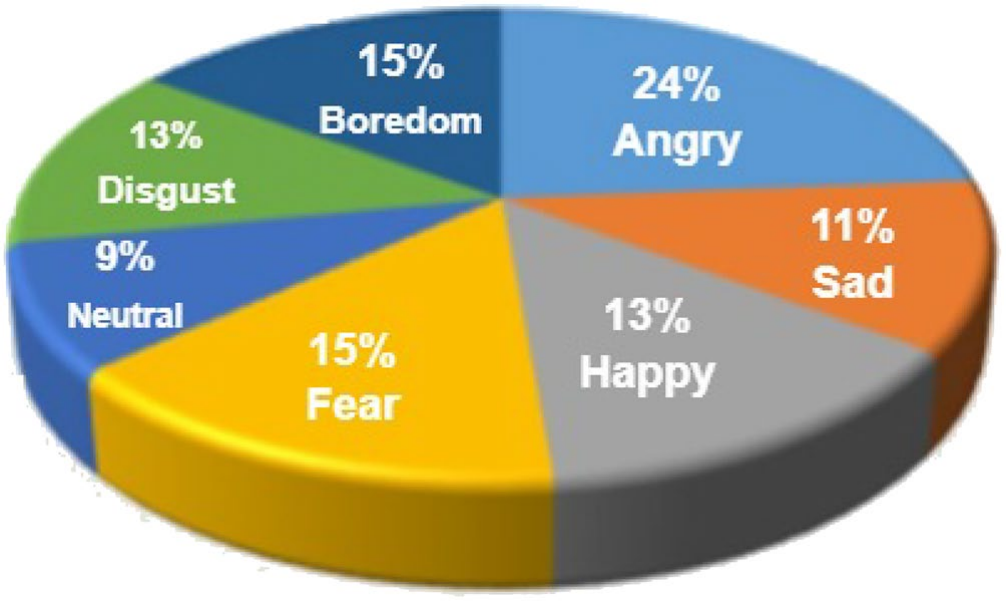
EMODB dataset emotion distribution.

#### EMODB

EMOD is one of the most predominantly utilized datasets, commonly known as the Berlin emotion dataset^66^ or the EMO-DB. This well-known and well-liked dataset of speech emotions contains 535 voice utterances expressing seven different emotions. Five men and five women, all experts, read prescriptive words and recorded various emotions for the suggested dataset. Time is captured with a sampling rate of 16 kHz and an average duration of 2 to 3 seconds in the EMO-DB corpus. Every utterance has the same temporal scale, allowing the entire speech to fit within the window size. The EMO-DB corpus, which is widely used in the SER field, forms the foundation for several emotion recognition algorithms. Figure 5 illustrates the summary of the overall utterances, participation rate, and selected emotions.

### Model implementation

The primary framework for the model implementation uses PyTorch^67^ components. We modified the size of the images during the pre-processing stage to accommodate the dimensions of 224*x*224 on three separate channels (corresponding to the RGB channels); we have delved into more depth about speech data pre-processing in the previous section. The experiment was carried out on a computing resource that includes $GPU-10900K@3.70 Ghz$, 64GB RAM, and the Google Colab platform. We utilized the Adam optimizer with sparse categorical cross entropy loss function and $3.63E-03$ as the learning rate during the training phase. We obtained optimum accuracy at 75 epoch. Finally, using a simple momentum of 0.9, we accelerated training and variable learning by the experiment’s chosen optimizer. Two public datasets (TESS and EMODB) are used, with the combination of the two datasets to form the third set of datasets (TESS-EMOD) for assessing the performance and generalizability of our model. The overall description of the hyperparameters utilized in this work is highlighted in Table 2

**Table 2:** Hyperparameters employed for this study.

Hyperparameter	Value
Number of Epochs	75
Learning rate	3.63E-03
Activation function	GELU
Embedded dropout rate	0.1
Trainable parameters	4,166,151
Patch size	32
MLP dimension	128
Optimizer	Adam
Loss Function	Flattened Loss of Cross Entropy

### Evaluation metrics

Standard metrics are typically used to evaluate the effectiveness of deep learning models for emotion identification tasks. Based on several performance criteria, including precision, recall, accuracy, and F1-score as provided in Eqs. (6)–(9), the proposed method’s results are contrasted. Precision and recall reflect the qualitative and quantitative performance of the proposed SER system, whilst accuracy represents the percentage of accurate predictions out of the total number of cases analyzed. Recall (sensitivity) measures the proportion of actual positive cases from all actual positive cases, while precision measures the proportion of true positive (TP) cases from all predicted positive cases. The harmonic mean of the precision and recall are provided by the F1-score^68^.

7 $$\begin{aligned} Precision = \frac{TP}{TP+FP} \end{aligned}$$

8 $$\begin{aligned} Recall = \frac{TN}{TN+FN} \end{aligned}$$

9 $$\begin{aligned} Accuracy = \frac{1}{N}\sum ^{N}_{i=1}\left( \frac{TP+TN}{TP+TN+FP+FN}\right) \end{aligned}$$

10 $$\begin{aligned} F1-Score = \frac{2\times Precision \times Recall}{Precision + Recall} \end{aligned}$$

Furthermore, we adopted the confusion matrix metric which gives a more meaningful insight into the outcome of our experiment. It uses variables such as FP (false positive), FN (false negative), TP (true positive), and TN (true negative)^69^ in depicting the combinations of true and predicted classes from a given speech dataset.

### Results of experiments and discussion

**Figure 6:**
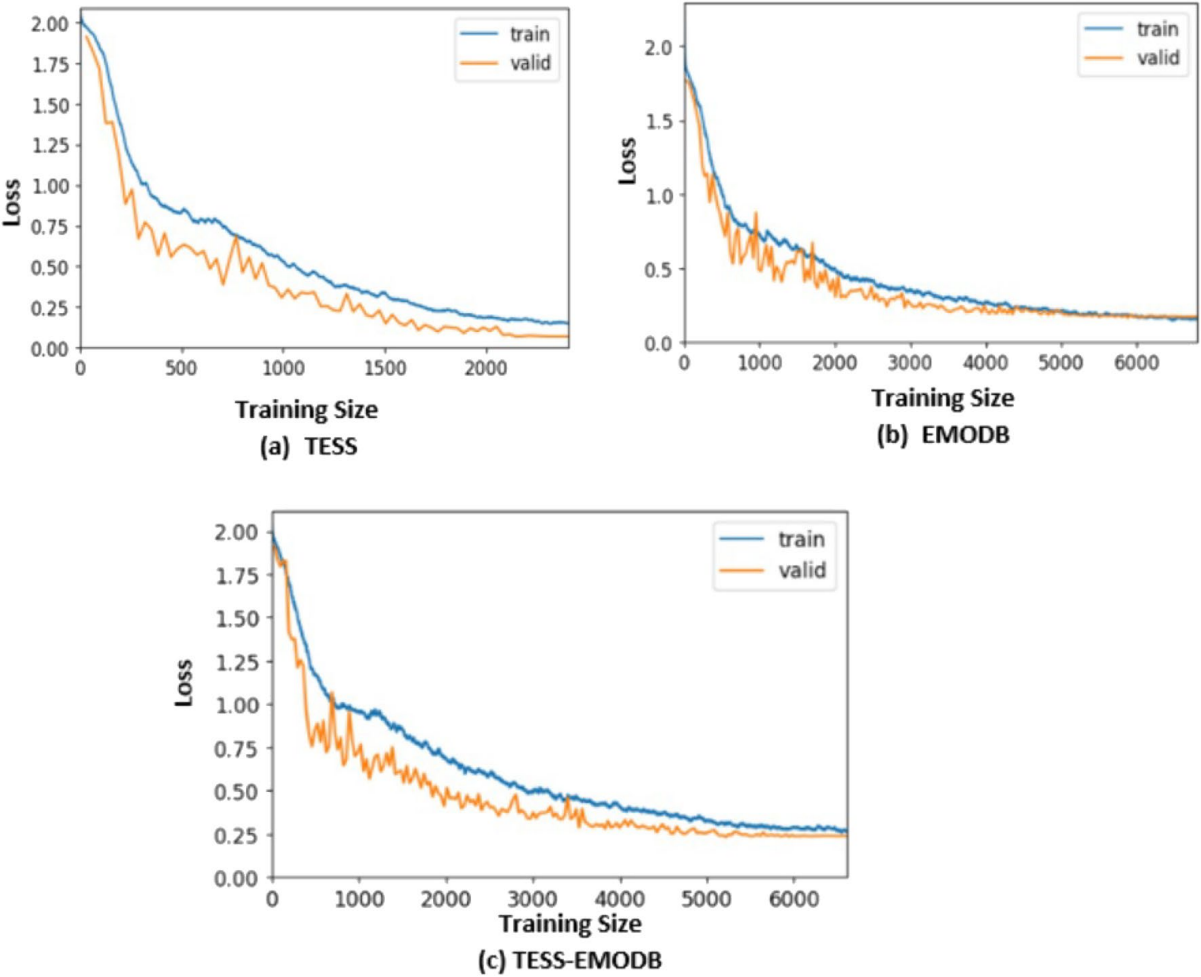
The figure illustrates the proposed model’s performance loss curve for the three benchmarked datasets. (**a**) Loss diagram on TESS dataset (**b**) Loss diagram on EMODB dataset and (**c**) Loss diagram on TESS-EMODB dataset.

This section describes the result of our extensive experiments carried out to assess the performance of our proposed model for speech emotion recognition tasks. The collection of tests is utilized to assess how well the model recognizes unknown speech utterances. The system generalization error is approximately represented by the model prediction error^70^. The cross-validation estimation approach is used in this study to thoroughly assess each dataset. The database’s data is divided into two categories: training data and testing data. There are k fragments to the original data in which the k part of the data is utilized for training, while one portion is used as test data. K-fold cross-validation is a term used to describe the test procedure, which is carried out k times across various portions of all the data^71^. For an in-depth assessment of our technique, we applied a well-known 5-fold cross-validation assessment method. The visual representation of the model loss is shown in Fig. 6. The uniqueness of our proposed model as displayed in the figure, indicates its effectiveness as the loss decreases on both training and testing data. The highest loss value for the three experiments were 0.13, 0.2 and 0.25 on TESS, EMODB and TESS-EMODB respectively.

According to the speech databases used, which include a variety of emotions-seven distinct ones-selected following Ekman’s^72^ postulation. We investigated the proposed model and presented the emotional level prediction performance in Tables 3, 4 and 5 together with the resulting confusion matrices. Our model’s prediction performance displays precision, recall, F1-Score, weighted results, and un-weighted results, which amply demonstrates the model’s superiority over state-of-the-art techniques. According to the detailed classification(emotional level prediction) report, it is obvious that the highest recognition was obtained for precision, F1-score and recall on neutral emotion with 100% from the TESS dataset, followed by disgust with 99% from EMODB respectively, and the least recall rate was recorded on boredom with 76%.

**Table 3:** Emotional level prediction for TESS dataset.

Emotion	Precision (%)	Recall (%)	F1-score (%)
Angry	100	98	99
Disgust	99	99	99
Fear	99	99	99
Happy	96	96	96
Neutral	100	100	100
Sad	100	98	99
Surprise	92	97	94
Accuracy	–	–	98
Weighted Average	98	98	98

**Table 4:** Emotional level prediction for EMODB dataset.

Emotion	Precision (%)	Recall (%)	F1-score (%)
Angry	90	96	92
Boredom	88	76	81
Disgust	99	93	96
Fear	95	85	89
Happy	82	89	85
Neutral	88	96	92
Sad	94	94	94
Accuracy	–	–	91
Weighted Average	92	91	91

**Table 5:** Emotional level prediction for TESS-EMODB dataset.

Emotion	Precision (%)	Recall (%)	F1-score (%)
Angry	87	96	91
Disgust	92	95	93
Fear	97	93	95
Happy	94	87	90
Neutral	97	92	94
Sad	100	99	99
Accuracy	–	–	93
Weighted Average	94	93	93

**Figure 7:**
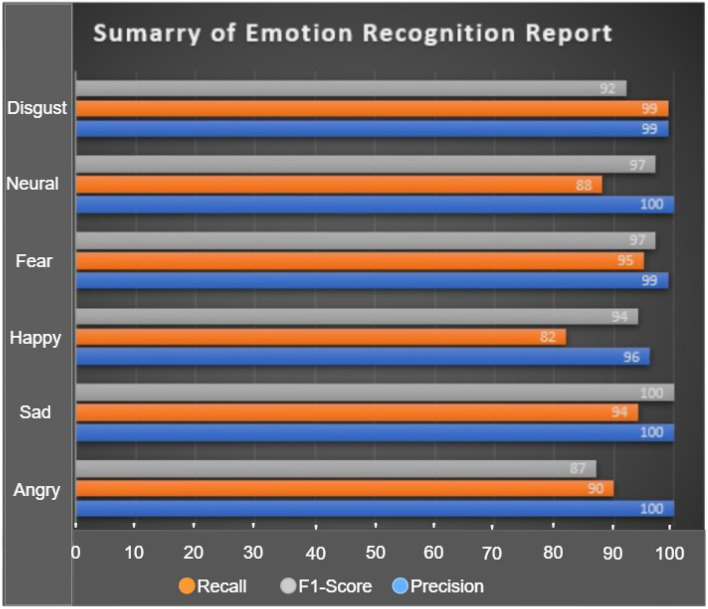
Summary of classification report for F1-Score, Recall and Precision.

We summarized the classification report in the above tables for each emotion using 3 metrics on 6 emotions as shown in Fig. 7. Our method demonstrates higher performance than the state-of-the-art approach in terms of the overall recognition of emotions, especially for disgust, neutral, sad and fear respectively. Our model recognizes the emotions from the frequency pixels and salient features to enhance recognition accuracy and mitigate the overall computational cost. Most of the baseline models detected disgust emotions with low accuracy because of their paralinguistic content, however, our model outperformed others with high precision and recall of 99% with only happy emotion demonstrating the least recognition of 82% recall.

**Figure 8:**
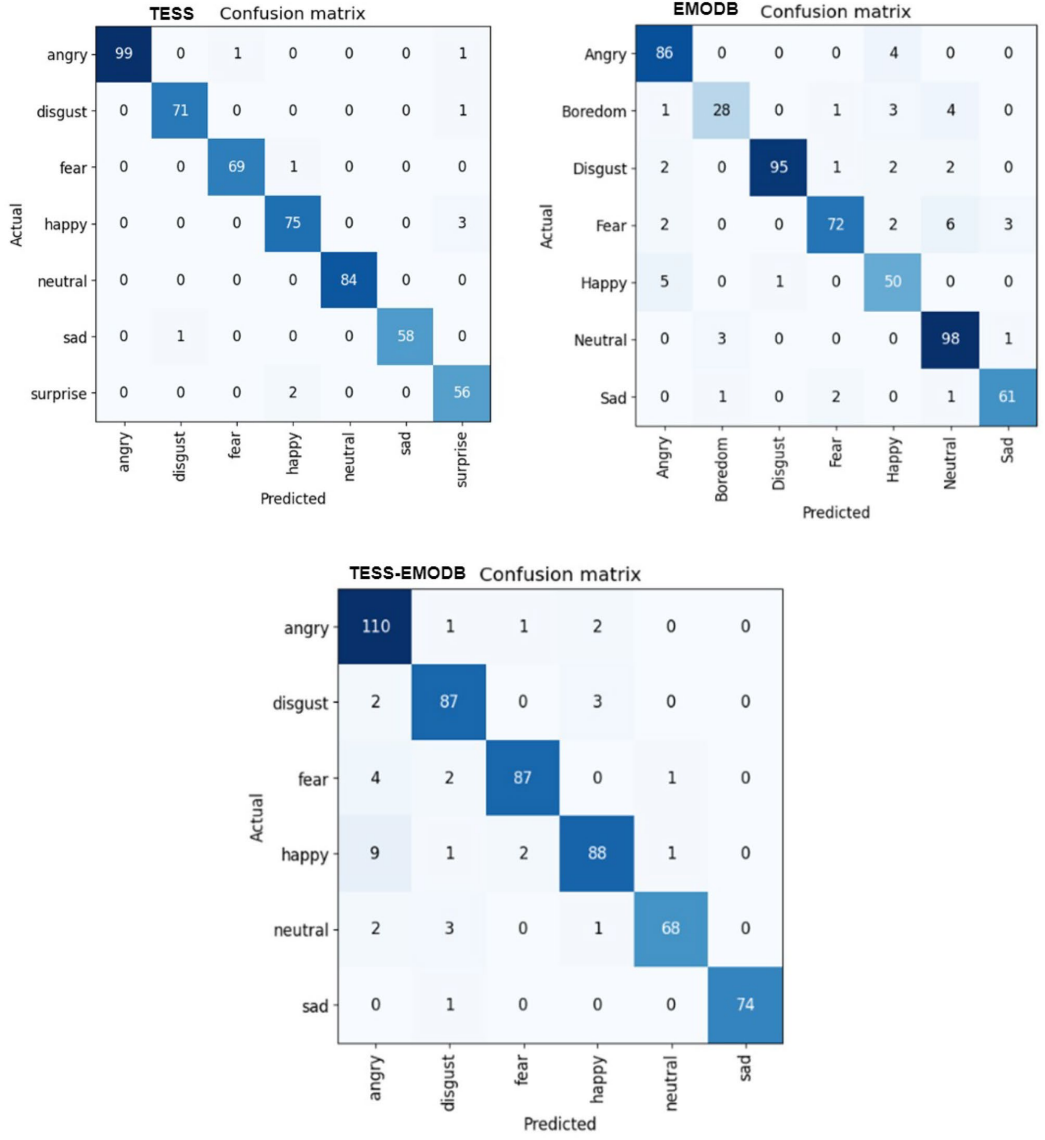
Confusion matrix for TESS, EMODB and TESS-EMODB.

**Figure 9:**
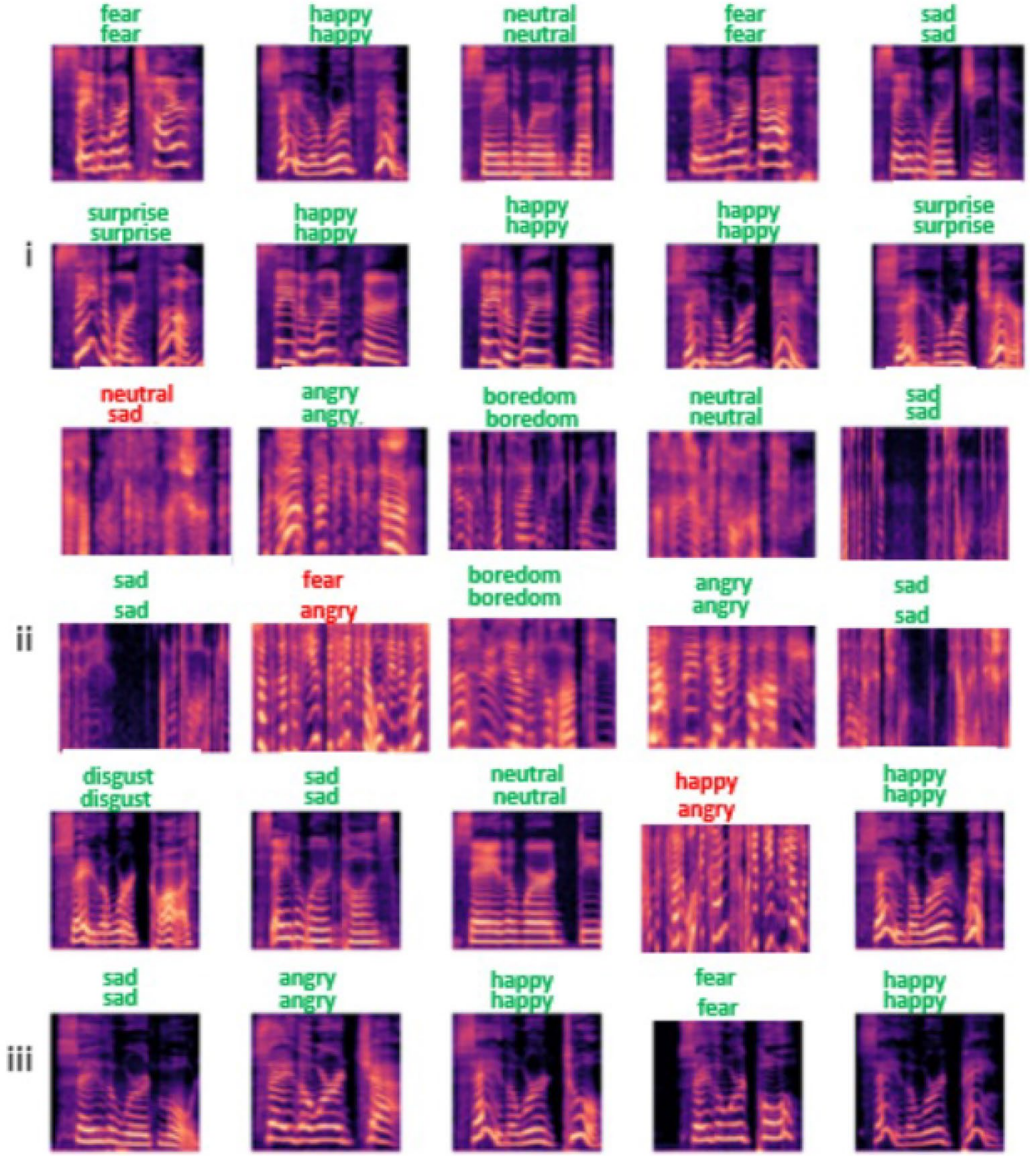
Test sample of emotion recognition output of the proposed model on three datasets: (i) represents recognition output on TESS dataset (ii) represents recognition output on EMODB dataset (iii) represent recognition output on TESS-EMODB dataset.

In furtherance of our investigation, we obtain a confusion matrix for the three datasets to show a class-wise recognition accuracy as shown in Fig. 8. We achieved the highest recognition accuracy from the confusion matrix on angry, neutral and disgust with 99%, 98% and 95% respectively. Only boredom emotion showed the least recognition from our confusion matrix, but the classification report recorded a vivid minimum recognition of 76.0% recall and 88.0% precision. The hybrid dataset of TESS-EMODB recorded the lowest accuracy 74% on sad emotion and a 100% overall for angry emotion for six emotions, which further established the robustness of our proposed model for SER.

The simplicity of the model architectural design has in doubt contributed to its performance in enhancing the SER recognition rate, thereby, reducing misclassification of emotion and making it suitable for real-time applications in monitoring human behavioural patterns. The novelty of the model inappropriately recognizing emotion from speech utterances(mel-spectrogram) is also confirmed with selected emotion as shown in Fig. 9. Only three emotions out of about thirty selected for the test had wrong predictions, but twenty-seven of the rest were rightly predicted as the actual emotion. The first label represents the actual emotion, while the second label directly under it is the predicted.

### Performance evaluation

The comparative experiment aimed to evaluate the exact role that the Vision Transformer (ViT) model contributed to enhancing the speech emotion recognition ability that we observed. To carry out this extensive experiment, we substituted other deep learning-based architectures for the ViT model in our proposed framework, as shown in Table 6.

**Table 6:** Deep learning architectures comparative experiments on EMODB and TESS Datasets.

Architectures	Number of Parameters	Dataset	Accuracy
ResNet	9,116,032	SDT1	87.12
		SDT2	83.90
MobileNet	4,806,855	SDT1	48.50
		SDT2	81.32
InceptionNet	9,604,544	SDT1	62.30
		SDT2	75.60
DenseNet	7,043,654	SDT1	86.13
		SDT2	79.24
**ViTSER**	**4,166,151**	**SDT1**	**91.03**
		**SDT2**	**98.00**

Bold highlights our proposed model and its results.

Though, while processing visual data in a similar way to the ViT model, they did not possess the distinctive architectural features of the ViT in capturing long-range dependencies efficiently. Two speech datasets used in this work are represented by the SDT1 and SDT2. The comparative study’s results, which showed that the ViT model could enhance speech emotion recognition with fewer parameters while still achieving higher accuracy than other architectures, provided significant fresh insight. The apparent decrease in accuracy when utilizing other architectures highlights the significance of the self-attention mechanism of the ViT model in detecting nuanced spatial relationships that are essential for comprehending emotional nuances in human speech.

The comparative analysis of our proposed model’s superior performance with other existing methods was carried out as illustrated in Table 10, using the selected speech emotion database, to demonstrate further our SER method’s generalizability and suitability for real-time applications. The proposed method demonstrates the recent success of deep learning transformer in the SER domain, which recognized all the emotions with high accuracy, including even the neutral emotion, using an unambiguous architecture. In the table, we reveal the surpassing results of the proposed system, which are substantially greater than other methods, indicating the efficiency of our method. We carried out ablation experiments as indicated in Tables 7, 8, 9, with a focus on various patch sizes of the spectrogram image and removal of the embedded dropout layer component of the proposed model. The first experiment result obtained from Table 7 shows that the removal of the embedded dropout layer as a functional component of the model significantly reduces the speech emotion recognition accuracy. The accuracy dropped by 6%, and 2.03% on TESS and EMODB datasets respectively. Likewise, the second ablation experiment’s results from the two datasets with varying patch sizes indicated that the model declined in overall accuracy(OVA) as the patch size decreased. However, 14 and 32 represent the minimum and maximum patch sizes utilized in the experiments(Tables 8 and 9). It was obvious during the experiment that patch sizes above 32 increase the computational complexity, therefore we stopped at 32 which yielded an optimum accuracy without any need for parameter tuning (Table 10).

**Table 7:** Ablation Experiment 2 on TESS and EMODB: Removal of dropout layer from the model architecture: *A* Angry, *H* Happy, *S* Sad, *D* Disgust, *N* Neutral, *F* Fear, *B* Boredom, *Sr* Surprise, *B* Boredom, *P* Precision, *R* Recall, *F1* F1-Score.

Dataset	A	D	F	H	N	S	Sr	OVA(%)
TESS	0.86	0.92	0.95	0.87	0.94	0.98	0.97	92
	0.90	0.93	0.89	0.86	0.95	0.98	0.95	
	0.88	0.92	0.92	0.86	0.94	0.98	0.96	
EMODB	A	B	D	F	H	N	S	
	0.88	0.86	0.85	0.91	0.92	0.84	0.96	89
	0.92	0.81	0.86	0.94	0.87	0.94	0.84	
	0.90	0.84	0.85	0.93	0.90	0.88	0.89

**Table 8:** Ablation Experiment 1 on various patch sizes of audio spectrogram representation with TESS dataset: *A* Angry, *H* Happy, *S* Sad, *D* Disgust, *N* Neutral, *F* Fear, *B* Boredom, *Sr* Surprise, *P* Precision, *R* Recall, *F1* F1-Score.

Size	Metrics	A	D	F	H	N	S	Sr	OVA(%)
14	P	0.83	0.90	0.93	0.90	0.92	0.98	0.97	91
	R	0.90	0.91	0.91	0.80	0.96	0.96	0.95	
	F1	0.86	0.91	0.92	0.85	0.94	0.97	0.96	
16	P	0.84	0.92	0.99	0.90	0.90	0.98	0.97	92
	R	0.94	0.96	0.88	0.82	0.97	0.91	0.98	
	F1	0.89	0.94	0.93	0.86	0.93	0.94	0.98	
28	P	0.86	0.96	0.98	0.90	0.95	0.98	0.98	94
	R	0.97	0.93	0.91	0.87	0.95	0.99	0.95	
	F1	0.91	0.94	0.95	0.88	0.95	0.98	0.97	
**32**	P	1.00	0.99	0.99	0.96	1.00	1.00	0.92	**98**
	R	0.98	0.99	0.99	0.96	1.00	0.98	0.97	
	F1	0.99	0.99	0.99	0.96	1.00	0.99	0.94	

Bold highlights our proposed model and its results.

**Table 9:** Patch size ablation experiment on EMODB dataset: B- Boredom.

Size	Metrics	A	D	F	H	N	S	Sr	OVA(%)
14	P	0.87	0.83	0.80	0.86	0.86	0.86	0.96	86
	R	0.94	0.74	0.86	0.93	0.81	0.87	0.81	
	F1	0.90	0.78	0.82	0.90	0.84	0.87	0.88	
16	P	0.85	0.78	0.90	0.93	0.80	0.74	0.91	92
	R	0.96	0.67	0.90	0.92	0.74	0.90	0.76	
	F1	0.90	0.72	0.90	0.93	0.77	0.81	0.83	
28	P	0.87	0.89	0.81	0.86	0.86	0.86	0.88	86
	R	0.93	0.63	0.88	0.90	0.86	0.88	0.84	
	F1	0.90	0.74	0.84	0.88	0.86	0.87	0.86	
**32**	P	0.90	0.88	0.99	0.95	0.82	0.88	0.94	**91**
	R	0.96	0.76	0.93	0.85	0.89	0.96	0.94	
	F1	0.92	0.81	0.96	0.99	0.85	0.92	0.94	

Bold highlights our proposed model and its results.

**Table 10:** Comparison with other baseline studies using TESS and EMODB dataset.

Year	Author & References	Method	Dataset	Accuracy (%)
2018	Chen et al.^73^	CNN+Attention	EMODB	82.82
2019	Jiang et al.^74^	CRNN	EMODB	84.49
2019	Meng et al.^75^	BiLSTM	EMODB	88.99
2020	Mustaqeem et al.^76^	CNN	EMODB	85.57
2020	Kwon,^77^	CNN	EMODB	90.01
2022	Guizzo et al.^78^	Quantarion CNN	EMODB	88.47
2022	Wen et al.^79^	Transfer Learning	EMODB	84.14
**2023**	**Proposed**	**ViTSER**	**EMODB**	**91.03**
2017	Verma, et al.^80^	SVM	TESS	96.00
2018	Praseetha et al.^81^	DNN	TESS	89.96.
2019	Gao^82^	CNN	TESS	81.00
2021	Krishnan et al.^83^	Decomposition	TESS	93.30.
2021	Chimthankar^84^	DNN	TESS	96.00.
2022	Akinpelu & Viriri^85^	VGGNet+RF	TESS	96.10.
2022	Guizzo et al.^78^	Quantarion CNN	TESS	97.00
2022	Choudhary et al.^86^	DNN	TESS	87.10
**2023**	**Proposed**	**ViTSER**	**TESS**	**98.00**

Bold highlights our proposed model and its results.

## Conclusion

In this research, a novel Vision Transformer model based on the mel-spectrogram and deep features was developed for the problem of speech emotion recognition. To assure accuracy, a simple MLP head attention with 128 dimensions was utilized to extract the deep features. With flattening, tokenizer, 32 patch size, position embedding, self-attention, and MLP head layers for enhancing SER, we developed a vision transformer model. The computational complexity was minimized due to the compactness of our model architecture, which is responsible for reducing an excessive number of parameters. To demonstrate the efficacy along with the significance and generalization of the model, its performance was assessed using two benchmark datasets: TESS and EMO-DB as opposed to^25^. The proposed system outperformed the state-of-the-art in terms of prediction results. Extensive experiments using our model produced astounding recognition accuracy scores of 98% for the TESS dataset, 91% for the EMO-DB, and 93% when the two datasets were combined. In order to recognize all emotions with better accuracy and a smaller model size to produce computationally friendly output, the proposed model improved by 2% and 5% over the state-of-the-art accuracy. The results of the proposed approach demonstrated the capability of Vision Transformer to capture global contextual information, making it possible to model long-range dependencies and enhance the representation of emotional speech patterns, ultimately leading to improved speech emotion recognition. We will concentrate on implementing this kind of system in additional speech recognition-related task systems in the future and go into more detail. Similar to this, we will conduct some tests to evaluate the effectiveness of the proposed method and the obtained results on other datasets, including non-synthetic speech corpora. When combined with other deep learning techniques, the recognition rates are likely to rise. Utilizing additional speech features such as the Mel-Frequency Cepstral Coefficient (MFCC), Chromagram, and Tonnetz can enhance the investigation as they form part of our future work as well.

## Data Availability

The two publicly available datasets used or analysed for this study are available at: (i) the Tspace repository (https://tspace.library.utoronto.ca/handle/1807/24487) for the TESS dataset and (ii) Berlin Database of Emotional Speech repository (http://emodb.bilderbar.info/showresults/index.php) for EMODB dataset.
